# Beyond borders: Do geographic correlations suggest shared environmental factors in inflammatory bowel diseases, Hodgkin lymphoma, and multiple sclerosis

**DOI:** 10.1002/ueg2.12428

**Published:** 2023-06-15

**Authors:** Shmuel Fay, Shomron Ben‐Horin

**Affiliations:** ^1^ Department of Gastroenterology Sheba Medical Center Tel HaShomer Israel; ^2^ Sackler Faculty of Medicine Tel‐Aviv University Tel Aviv Israel

**Keywords:** Crohn's disease, epidemiology, IBD, inflammatory bowel disease, ulcerative colitis

The etiology of inflammatory bowel diseases (IBD) is multifactorial and is believed to result from an interplay of inappropriate immune response, gut microbiota, genetic susceptibility, and environmental factors.^1^

In the current issue of the Journal, Amnon Sonnenberg used epidemiological tools to explore potential clues pertaining to the etiology of IBD. The author analyzed the death rates in four diseases—Hodgkin lymphoma (HL), multiple sclerosis (MS), Crohn's disease (CD), and ulcerative colitis (UC)—in 21 countries from 1951 to 2020, using World Health Organization vital records. In general, the death rates from these four diseases tended to be high in most European countries and low for most non‐European countries, and their associated death‐rates were correlated and characterized by similar geographic distributions, which were all statistically significant.

The author concluded that the remarkable similarity in geographic distribution implies the potential impact of shared environmental factors on the occurrence of these four diseases. Since typically, environmental risk factors have a universal effect, impacting all demographic segments of the population similarly, the author executed a second analysis for the age‐specific death rates of the individual countries, correlated with each other. This analysis revealed that In HL and UC, the inter‐age correlations started at age 5 years or less. In MS and CD, the inter‐age correlations only started at age 15 years.

These correlations suggest that the four diseases may share one or more common environmental risk factors. Such a conclusion could have far‐reaching implications for how we understand disease etiology, potentially guiding future research into prevention and treatment strategies.

The link between MS and inflammatory bowel diseases is well recognized.^2^ MS was associated with a 55% increased risk of IBD, and reciprocally, IBD patients had a 53% increased risk of MS.^2^ Genetic overlap was also described between these diseases.^3^ The linkage between IBD and HL is less established, although inflammatory mechanisms are part of HL pathogenesis.^4^, ^5^ EBV plays a major role in HL pathogenesis and perhaps also in MS, but is not a known predisposing factor in IBD.

However, despite this innovative methodology and mind‐provoking findings, some limitations need to be acknowledged. The study spans a compelling temporal range of 70 years, but this also opens the possibility of various biases and confounders, such as changes in diagnostic criteria, improvements in healthcare access, and differing reporting practices over time, which could all influence reported death rates. All four diseases were found correlated, and the lack of a negative control in the form of a disease tested by the same methodology and found uncorrelated with the other four stands out as yet another limitation. Finally, reliance on death rates is a well‐documented surrogate marker of disease prevalence, but the advent of advanced medical treatments over the period covered by the study has likely reduced mortality rates, possibly differently between territories.

The study does support previous knowledge demonstrating the influence of environmental factors on inflammatory bowel diseases. Past research has indicated that distinct environmental factors manifest differently in their impact on inflammatory bowel diseases in Western versus Eastern regions of the world.^6^ The under‐representation of Asian and African populations in the present databases may limit the generalizability of findings. Indeed, EBV has been shown to affect Asian populations at an earlier age with over 90% sero‐prevalence at age 5, as compared with Western populations in whom this rate is reached by age 22^7^, thereby arguing against EBV as an environmental risk factor explaining the higher IBD rates in the West.

In conclusion, this study represents a significant step forward in the epidemiological study of HL, MS, CD, and UC, and paves a conceptual path for further research. Nuanced outcome metrics, broader geographic representation, and a deep investigation into the shared environmental risk factors of IBD vis‐à‐vis other diseases may reveal unsuspected environmental factors responsible for the surge in IBD incidence in recent decades.

## FUNDING INFORMATION

This Editorial received no specific grant from any funding agency in the public, commercial, or not‐for‐profit sectors.

## CONFLICT OF INTEREST STATEMENT

The authors have no conflicts of interest to declare.

## Data Availability

The data underlying this article will be shared on reasonable request to the corresponding author.
